# Visible Foliar Injury and Ecophysiological Responses to Ozone and Drought in Oak Seedlings

**DOI:** 10.3390/plants11141836

**Published:** 2022-07-13

**Authors:** Barbara Baesso Moura, Elena Paoletti, Ovidiu Badea, Francesco Ferrini, Yasutomo Hoshika

**Affiliations:** 1 Institute of Research on Terrestrial Ecosystems (IRET), National Research Council of Italy (CNR), Via Madonna del Piano 10, 50019 Sesto Fiorentino, Italy; bmourabio@gmail.com (B.B.M.); yasutomo.hoshoka@unifi.it (Y.H.); 2 “Marin Drăcea“ National Institute for Research and Development in Forestry, 128 Eroilor Blvd., 077190 Voluntari, Romania; ovidiu.badea63@gmail.com; 3 Faculty of Silviculture and Forest Engineering, “Transilvania” University of Brasov, 1, Ludwig van Beethoven Str., 500123 Braşov, Romania; 4 Department of Agriculture, Food, Environmental and Forestry Sciences, Section Woody Plants, University of Florence, 50019 Sesto Fiorentino, Italy; francesco.ferrini@unifi.it

**Keywords:** tropospheric ozone, leaf symptoms, PODy, water stress, risk assessment

## Abstract

To verify the responses of visible foliar injury (VFI), we exposed seedlings of three oak species for 4.5 months in an open air facility, using differing ozone (O_3_) and drought treatments: O_3_ (three levels from ambient to ×1.4 ambient), and drought (three levels of irrigation from 40% to 100% field capacity). We related the accumulated phytotoxic O_3_ dose (POD_1_) and cumulative drought index (CDI) to the O_3_ and drought VFI and assessed growth increment (height, diameter, leaf number), biomass (of all organs), and physiological parameters: net photosynthesis per plant (*P*_n_), photosynthetic nitrogen (PNUE) and phosphorus use efficiency (PPUE)). The results indicated that an increase in POD_1_ promoted O_3_ VFI in *Quercus robur* and *Quercus pubescens,* while *Quercus ilex* was asymptomatic. The POD_1_-based critical level at the onset of O_3_ VFI was lower for *Q. robur* than for *Q. pubescens* (12.2 vs. 15.6 mmol m^−2^ POD_1_). Interestingly, drought reduced O_3_ VFI in *Q. robur* but increased it in *Q. pubescens*. Both O_3_ and drought were detrimental to the plant biomass. However, *Q. robur* and *Q. pubescens* invested more in shoots than in roots, while *Q. ilex* invested more in roots, which might be related to a hormetic mechanism. *P*_n_, PNUE and PPUE decreased in all species under drought, and only in the sensitive *Q. robur* (PPUE) and *Q. pubescens* (PNUE) under O_3_. This study confirms that POD_1_ is a good indicator to explain the development of O_3_ VFI and helps a differential diagnosis of co-occurring drought and O_3_ VFI in oak forests.

## 1. Introduction

Tropospheric ozone (O_3_) is an oxidative pollutant harmful to plants [1]. Ozone enters the leaves through the stomata, reacts in the mesophyll, and triggers the formation of reactive oxidative species (ROS) with a cascade of events eventually promoting cell death and, finally, the appearance of visible foliar injury (VFI), physiological impairment, and growth reduction [2,3,4]. Furthermore, O_3_ inhibits the efficient use of nutrients such as nitrogen (N) and phosphorus (P) and thereby causes a reduction of photosynthetic N and P use efficiency (PNUE and PPUE, respectively) [5,6]. Therefore, critical levels (CL) have been investigated to assess the O_3_ negative impacts on several plant species, especially those related to biomass loss [7,8]. CLs are based on cumulative O_3_ indexes, e.g., AOT40, defined as the accumulated exposure over 40 ppb hourly concentrations, and PODy, defined as the phytotoxic O_3_ dose above an hourly threshold y of stomatal O_3_ uptake [9]. PODy is considered the most realistic index with a high correlation with the detrimental effects of O_3_ [10,11]. Ozone VFI is a forest-health indicator in forest monitoring programs [12]. The estimation of CL based on O_3_ VFI has been proposed as a not destructive and easily repeated observation over long-term monitoring studies [13,14].

Ozone alone can affect plant growth and development, but its effect usually occurs in combination with other factors, such as drought, which is known as the most critical environmental factor limiting plant productivity worldwide [15,16]. The adverse effects of drought are progressive and, thus, are often evaluated by the cumulative drought index (CDI), defined as the accumulated difference of soil moisture relative to field capacity [17]. Drought stress also promotes the formation of specific VFI, which can be distinguished from O_3_-induced foliar injury. While O_3_ VFI is usually indicated by interveinal, irregular-border, yellow to dark-brown stippling [18,19], drought VFI consists in gradients of leaf margin necrosis increasing in severity from the base to the top of a plant [20], with the injury co-occurring when plants are exposed to a combination of these stress factors. 

Both O_3_ and drought can limit plant carbon fixation, and the effect of both stress factors has been reported as the cause of biomass loss for *Quercus* species [21,22], which are significant components of temperate forests. Previous papers from the same experiment presented here showed that the interacting factorial impacts of O_3_ and drought were species-specific, and the order of O_3_ sensitivity was *Q. robur* > *Q. pubescens* > *Q. ilex* from the point of view of total biomass [22] and leaf gas exchange [23,24]. Although physiological acclimations to O_3_ and drought are not fully elucidated, diverse adaptation strategies were observed for tolerating stress in different oak species. One of the reasons for the variability of strategies is related to gas exchange regulation depending on their water use strategy (isohydric and anisohydric) [24]. Under elevated O_3_ with sufficient water availability, the isohydric *Q. robur* limited O_3_ uptake by stomatal closure, while the anisohydric *Q. ilex* and the intermediate *Q. pubescens* activated tolerance mechanisms and did not actively show a closing response of stomata. In particular, Pellegrini et al. [25] found that *Q. ilex* had a well-regulated antioxidative defense system through phenylpropanoid pathways. However, in the combination of O_3_ and drought, the anisohydric *Q. ilex* and the intermediate *Q. pubescens* exhibited stomatal closure to prevent severe oxidative damage due to excess generation of ROS. 

The present study aimed to characterize the VFI induced by O_3_, drought, and their combination and assess their related effects on biomass, biometry, and physiological parameters. The results will help a differential diagnosis of co-occurring drought and O_3_ VFI in oak forests. In detail, we addressed the following hypotheses: (1) the development of O_3_ VFI may be better explained by PODy than by AOT40, (2) the reduction in soil water availability may reduce or exacerbate the negative impacts of O_3_ on VFI, and (3) O_3_ VFI may be an indicator to explain biomass reduction or physiological damage in Mediterranean oaks. We postulated that the effects on the development of VFI are modulated by the plant species-specific sensitivity to oxidative stressors.

## 2. Materials and Methods

### 2.1. Plant Material and Experimental Setting

The experiment was conducted in an O_3_ Free-Air Controlled Exposure (FACE) facility at Sesto Fiorentino, Italy (43°48′59″ N, 11°12′01″ E, 55 m a.s.l.). Two-year-old plants of *Q. robur* L., *Q. pubescens* Willd., and *Q. ilex* L. were obtained from nurseries and transplanted into 10-L plastic pots. They were exposed to three levels of O_3_ (1.0, 1.2, and 1.4 times the ambient air concentration, denoted as AA, ×1.2, and ×1.4, respectively: 24-h averaged concentration, AA = 35.2 ppb, ×1.2 = 42.9 ppb, ×1.4 = 48.9 ppb) and three levels of water irrigation [100, 80, and 40% of field capacity (0.295 m^3^ m^−3^, Paoletti et al., 2017) on average, denoted as WW-treated (well-watered), MD-treated (moderate drought) and SD-treated (severe drought), respectively]. Three replicated plots were assigned to each treatment, with three plants per combination of species, drought, and O_3_. The experiment lasted for 4.5 months, from 1 June to 15 October.

The details of the FACE facility are described in Paoletti et al. [26], and the details of the experimental design are published in Hoshika et al. [27].

### 2.2. Evaluation of O_3_ and Drought Visible Foliar Injury

Two well-trained observers evaluated the presence of O_3_ and drought VFI during the experimental period for all plants for a total of 6 evaluation dates (Table 1). We applied photo guides to verify whether O_3_ and/or drought VFI was present [28,29,30]. VFI incidence (INC = number of injured plants/total number of plants × 100) was calculated according to Chappelka et al. [31]. POD_1_-based CLs and CDI-based CLs were calculated for the corresponding day when O_3_ and drought VFI onset was observed.

### 2.3. Measure of Growth Parameters

The assessment of total annual biomass production during the experiment was performed based on dry weight per plant (DW) as described in Hoshika et al. [22], additionally discriminating the below-(roots) and above-ground biomass (stem and leaves) to calculate the ratio of root to shoot biomass (Ratio R/S). Furthermore, the total number of leaves, plant height increment (measured with a metric tape) and stem caliber increment (measured just above soil level) were expressed as the absolute values relative to the values at the beginning and end of the experiment.

### 2.4. Assessment of Photosynthetic Parameters

The net photosynthetic rate (*P*_n_) was previously reported for mid-summer (July: [24]) and early and late summer and autumn (June, August, and October: [23]). Here, these published data of *P*_n_ were re-analyzed to address the cumulative effects of O_3_ and drought on the photosynthetic activity. The target leaves were fully sun-exposed leaves (4–6th from the shoot tip) of the plant main shoot (one representative leaf per plant, 1 to 3 plants per replicated plot per each O_3_ and W treatment). Measurements were made under light-saturated conditions (1500 μmol m^−2^ s^−1^ PPFD [photosynthetic photon flux density]) with constant CO_2_ concentration (400 μmol mol^−1^), relative humidity (40 to 50%), and leaf temperature (25 °C) using a commercial gas exchange system (CIRAS-2 PP Systems, Herts, UK). Measurements were carried out in two campaigns (8–10 June and 27 September–6 October) for all O_3_ treatments and an additional campaign (6–9 August) for two O_3_ levels (1.2, and ×1.4) on days with clear sky between 9:00 and 12:00 a.m. CET. The other detailed specifications for the photosynthetic measurements were described in our previous studies [23,24].

After the measurement of *P*_n_ in August and October, leaves were collected to examine the nitrogen (N) content. Nitrogen content per unit mass (N_mass_) was determined by the dry combustion method using a LECO TruSpec C/N analyzer (Leco Corporation, St. Joseph, MI, USA). In October, the foliar phosphorus (P) content was also determined. Phosphorus content per unit mass (P_mass_) was examined by an inductively coupled plasma-optical emission spectroscopy (ICP-OES) (iCAP7000, Thermo Fisher Scientific, Waltham, MA, USA). We calculated photosynthetic N use efficiency (PNUE) as the product of N_mass_ and mass-based net photosynthetic rate and photosynthetic P use efficiency (PPUE) as the product of P_mass_ and mass-based net photosynthetic rate.

### 2.5. Calculation of Accumulated Drought and Ozone Indexes

The accumulated drought index (CDI) was calculated from the beginning of the experimental period to the date of observation as follows:

$$\mathrm{CDI}=\sum \left|Sm-Fc\right|$$

where, *Sm* is soil moisture, and *Fc* is field capacity (0.295 m^3^ m^−3^) [26]; drought stress is considered severe when *Sm* values are lower than *Fc*.

AOT40 and POD_1_ for each O_3_ and drought treatment were calculated following the parameters applied by Hoshika et al. [22] according to the methodology designed by CLRTAP (Convention on Long-range Transboundary Air Pollution) [9].

### 2.6. Statistical Analysis

Multiple Linear Regression (MLR) analysis was used to estimate the relationship between the O_3_ indexes (AOT40 and POD_1_) and CDI versus growth (height, diameter, and N. leaves), biomass (Leaf, Shoot, Root, Total, and R/S), VFI (O_3_ and Drought) and physiological parameters (*P*_n_, PNUE, and PPUE). Two models were compared, i.e., Model 1 (POD_1_ and CDI as predictor variables) and Model 2 (AOT40 and CDI as predictor variables). The statistical analyses were performed using the R software (R version 4.1.2 [32]), considering a significance of *p* < 0.05. Principal component analysis (PCA) was conducted by using OriginPro 2021b software. The PCA was applied considering VFI (O_3_ and drought), growth (Height, Diameter, and N. of leaves), biomass (Leaf, Shoot, Root; Total and R/S), and physiological (Pn, PNUE, and PPUE) parameters in order to distinguish the groups of parameters better related to each symptomatic species; in this analysis, the asymptomatic *Q. ilex* species was not included.

## 3. Results

### 3.1. Visible Foliar Injury 

**Figure 1:**
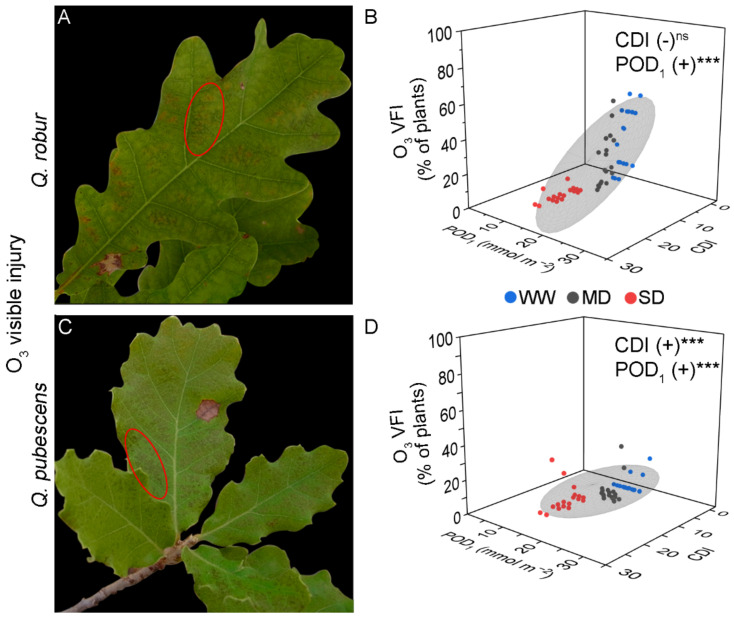
Illustrative examples of O_3_ visible foliar injury in *Quercus robur* (**A**) and *Q. pubescens* (**C**) characterized by small homogeneously distributed dots between the primary leaf veins (ellipse). (**B**,**D**) Results from the linear multiple regression of O_3_ visible foliar injury with Cumulative Drought Index (CDI) and phototoxic O_3_ dose (POD_1_) as predictor factors in *Quercus robur* (**B**) and *Q. pubescens* (**D**). Colored dots represent well-watered (WW-blue), moderate drought (MD-grey), and severe drought (SD-red). The grey ellipsoid represents a confidence level of 75%. (+) positive regression, *** = *p* < 0.001, ns = not significant.

The O_3_ VFI in *Q. robur* was characterized by small homogeneously distributed dots between the primary leaf veins (Figure 1A). *Q. robur* plants from all water regimes but SD (AA and ×1.2) presented O_3_ VFI (Table 1 and Table 2). In fact, 11% of the SD-treated plants developed O_3_ VFI at the end of the experiment, relative to 56% of the WW-treated plants (Table 2). 

There were individual-specific differences on the day of VFI onset. The POD_1_ values calculated for the O_3_ VFI onset in *Q. robur* were similar across O_3_ treatments (approximately 10.7 to 13.0 mmol m^−2^ POD_1_, average = 12.1 mmol m^−2^ POD_1_), while the AOT40 values corresponding to the O_3_ VFI onset increased from 15-16 ppm h to 26.2 ppm h; for SD-treated plants, the O_3_ VFI onset occurred only in ×1.4 (10.7 ppm h, Table 1). In addition, the MLR revealed a positive regression of O_3_ VFI with POD_1_ or AOT40 and a negative regression with CDI when tested with AOT40 (Model 2), but the effect was not significant when tested with POD_1_ (Model 1) (Figure 1B; Table 3).

The O_3_ VFI in *Q. pubescens* was similar to that developed by *Q. robur* (Figure 1C). Independently of the water regime, plants from AA did not show O_3_ VFI, while plants from ×1.2 and ×1.4 treatments presented O_3_ VFI (Table 1 and Table 2). The percentage of plants presenting VFI was lower than for *Q. robur,* with a maximum of 33% presenting VFI (Table 2). VFI occurred for the first time at DOY 247 or 266, i.e., around the end of the experiment. The POD_1_ and AOT40 values for the O_3_ VFI onset were 12.8–20.46 mmol m^−2^ POD_1_ (average = 16.8 mmol m^−2^ POD_1_) and 24–33 ppm h AOT40, respectively (Table 1). The MLR revealed a positive regression with the O_3_ indexes (POD_1_ and AOT40; Figure 1D, Table 3). Interestingly, CDI positively affected the O_3_ VFI when tested with POD_1_ (Model 1), but the effect was not significant when tested with AOT40 (Model 2) (Table 3).

**Figure 2:**
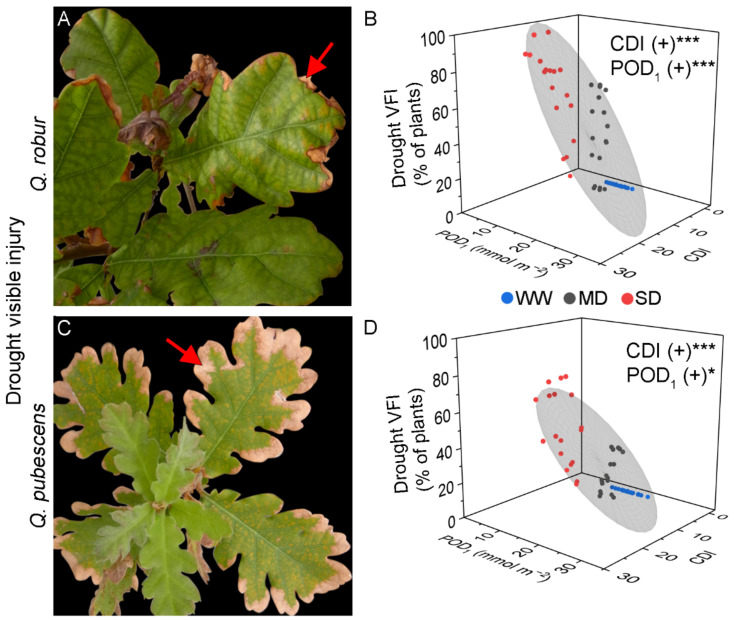
Illustrative examples of drought visible foliar injury in *Q. robur* (**A**) and *Q. pubescens* (**C**) characterized by dry and brownish leaf edges (arrow). (**B**,**D**) Results from the linear multiple regression of drought visible foliar injury with Cumulative Drought Index (CDI) and phototoxic O_3_ dose (POD_1_) as predictor factors in *Q. robur* (**B**) and *Q. pubescens* (**D**). Colored dots represent well-watered (WW—blue), moderate drought (MD—grey), and severe drought (SD—red). The grey ellipsoid represents a confidence level of 75%. (+) positive regression, * = *p* < 0.05, *** = *p* < 0.001, ns = not significant.

The drought VFI of *Q. robur* was evident exclusively on the leaf edge that became dry and brownish (Figure 2A). The VFI progressively increased in MD- and SD-treated plants until the end of the experimental period, while WW-treated plants did not show any injury (Table 2). At the end of the experiment, 89–100% of the SD-treated plants showed drought VFI, relative to 63–67% of the MD-treated plants (Table 2). The CDI calculated at the drought VFI onset was the same for all SD-treated plants (CDI = 10.20 for all AA, ×1.2, ×1.4 treatments, Table 1) and similar for MD-treated plants (CDI= 4.96 to 6.10, Table 1). The MLR revealed a strong positive regression between the *Q. robur* drought VFI and CDI, although POD_1_ or AOT40 also increased the extent of drought VFI (Figure 2B, Table 3).

The drought VFI of *Q. pubescens* was similar to that developed by *Q. robur* (Figure 2C). At the end of the experimental period, *Q. pubescens* presented 44–78% of the SD-treated plants with VFI, 11–33% of the MD-treated plants, and no VFI for the WW-treated plants. As found in *Q. robur*, the CDI calculated at the drought VFI onset was the same for all SD-treated plants (CDI = 10.20 for all AA, ×1.2, ×1.4 treatments) and similar for MD-treated plants (CDI= 4.04 to 6.52, Table 1). Interestingly, the CDI values at drought VFI onset were similar in *Q. robur* and *Q. pubescens* within the same O_3_ and drought treatments (Table 1). In addition, the MLR revealed a positive regression with CDI, POD_1,_ and AOT40 (Table 3, Figure 2D). The evergreen *Q. ilex* did not present O_3_ or drought VFI. 

### 3.2. Physiological Responses

In both *Q. robur* and *Q. pubescens*, *P*_n_ was negatively affected by POD_1_ and CDI (Table 3, Figure S1B and D), but it unexpectedly increased with increasing AOT40 (Table 3). Furthermore, PNUE and PPUE were negatively related to CDI and POD_1_ except for PNUE in *Q. robur* (Table 3).

For *Q. ilex,* the MLR revealed that CDI negatively affected *P*_n_ (Figure S1F), PNUE, and PPUE, with no significant relationship with the O_3_ indexes (POD_1_ and AOT40, Table 3). 

### 3.3. Growth and Biomass

The MLR indicated that height increment was positively affected by POD_1_ or AOT40 in *Q. robur*, while increments of diameter and number of leaves were negatively affected by CDI (Table 3). As confirmed by negative regression coefficients with CDI, most biomass parameters of *Q. robur* were reduced by drought. On the other hand, POD_1_ or AOT40 positively affected leaf biomass and negatively affected root biomass, indicating a reduction of the R/S ratio under elevated O_3_ exposure (Table 3, Figure S1A).

In *Q. pubescens,* the O_3_ indexes (POD_1_ and AOT40) were positively related to plant height increment, while CDI was negatively related to height only when tested with AOT40 (Table 3). Increments in shoot diameter and number of leaves in this species were negatively related to POD_1_ and AOT40, while they were negatively related to CDI when tested with POD_1_ (Model 1, Table 3). Regarding the biomass parameters, leaf biomass was not affected by any factor, while shoot biomass was negatively affected by both O_3_ indexes (POD_1_ and AOT40) and CDI (Table 3). Root and total biomass were negatively related to CDI, and the R/S ratio was negatively influenced by CDI and POD_1_ (Table 3, Figure S1C).

In *Q. ilex,* plant height increment was not affected by any factors, while a positive relationship between diameter increments and O_3_ indexes was found (Table 3). The increment in the number of leaves was positively affected by POD_1_ and AOT40 and positively affected by CDI when tested together with POD_1_ (Table 3), although leaf and total biomass were not significantly affected by those factors. Shoot biomass was negatively affected by CDI only when tested with AOT40, and root biomass was positively affected only by POD_1_ (Table 1). The R/S ratio was positively related only to POD_1_ (Table 3, Figure S1E).

The raw data off all growth parameters for the species *Q. robur*, *Q. pubescens*, and *Q. ilex* are avaible in Table S1.

### 3.4. Principal Component Analysis

**Figure 3:**
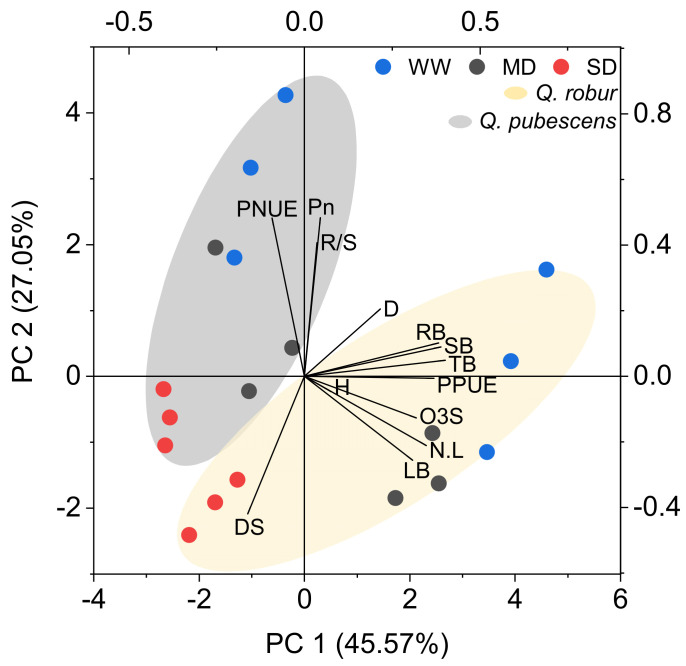
Bi-plot of the principal component analysis on Growth: Plant height increment (H), Stem diameter increment (S), and leaf number increment (N.L); Biomass: Leaf (L), Shoot (S), root (R), Total biomass (TB), and shoot/root ratio (R/S); Visible foliar O_3_ (O_3_S) and drought injury (DS); Physiological parameters: Photosynthesis (*P*_n_), Photosynthetic nitrogen use efficiency (PNUE) and Photosynthetic phosphorus use efficiency (PPUE).

The PCA detected separate multivariate spaces between the two symptomatic species as groups related to different growth, biomass, and physiological parameters related to O_3_ or drought VFI (Figure 3). 

Since *Q. ilex* did not show VFI, this species was not included in the analysis. The first two components of the PCA explained 45.57 and 27.05% of the variances. The SD-treated plants of both species were grouped near the drought VFI (DS) with no other parameter following the same vector direction. The individuals of *Q. robur* (especially MD-treated plants) were grouped near the vectors of the growth parameters number of leaves and height, leaf biomass, and O_3_ VFI, which presented the same direction, thus indicating that when O_3_ VFI increased, these parameters also increased. The individuals of *Q. pubescens* (specially WW-treated plants) were grouped near *P*_n_, PNUE, and R/S, with the vectors in the opposite direction of O_3_ and drought VFI, thus indicating that when O_3_ and drought VFI increased, these parameters decreased. 

## 4. Discussion

### 4.1. Development of Visible Injury Due to Ozone and Drought Stress

The POD_1_ values corresponding to the onset of O_3_ VFI for the two symptomatic deciduous oaks (on average, 14.4 mmol m^−2^) were similar to those estimated for broadleaf species under field conditions in Italy and France (10 mmol m^−2^ s^−1^) [10]. However, the CL for the VFI onset was lower for *Q. robur* than for *Q. pubescens,* indicating its higher sensitivity to O_3_, possibly related to its lower antioxidative capacity and inability to protect the cell structure [25]. Furthermore, O_3_ VFI increased with increasing POD_1_ in the two deciduous oaks. This suggests that PODy is a key indicator to describe the development of O_3_ VFI [33] once it is well known that O_3_ damage is closely related to stomatal O_3_ uptake [1]. In fact, the absence of O_3_ VFI in *Q. ilex* might be related to its low *g*_max_ (165 mmol O_3_ m^−2^ s^−1^, compared to 225 mmol O_3_ m^−2^ s^−1^ and 200 mmol O_3_ m^−2^ s^−1^ of *Q. pubescens* and *Q. robur,* respectively, [22] suggesting that the development of VFI might be discussed in terms of the specific-species patterns of stomatal conductance.

For both injured species (*Q. robur* and *Q. pubescens*) at the end of the experimental period, the severe drought treatment reduced POD_1_ by 30 to 40% [22], which would be expected to decrease the O_3_-induced VFI in plants as reported before for ecophysiological responses in poplars [34]. In *Q. robur,* the presence of O_3_ VFI was decreased under drought. On the contrary, drought stress aggravated the O_3_ VFI in *Q. pubescens*. Drought has been reported to have the potential to aggravate the harmful effects of O_3_ [35]. Furthermore, Hoshika et al. [24] found that the combination of O_3_ and drought altered the activity of the antioxidant system so that *Q. pubescens* was not protected from the severe oxidative stress resulting from the combined stress of O_3_ and drought.

For the symptomatic species (*Q. robur* and *Q. pubescens*), the progression of drought VFI could be attributed to the obstruction of conducting tissue [20], conferring to both species a high sensitivity. In the asymptomatic *Q. ilex,* this phenomenon might not happen due to its capacity to increase the cell wall thickness by reinforcing the strength and rigidity of the secondary cell walls with hemicellulose and lignin deposition (data not published). Changes in lignin might function as physical desiccation tolerance and maintenance of protein integrity in drought-tolerant species [36], thus helping the photosynthetic recovery activity after re-watering from severe drought episodes [37]. The CDI threshold for the appearance of drought VFI in the two symptomatic species was higher in SD-treated than MD-treated plants, possibly due to the interaction with leaf aging, which is an important physiological and biochemical defense factor against drought stress [38]. In fact, most plants showed drought VFI in mid- or late-summer in both SD-treated and MD-treated plants when leaves were relatively old.

### 4.2. Effects of Ozone and Drought Stress on Growth and Biomass Parameters

For both deciduous species (*Q. robur* and *Q. pubescens*), height increment was higher when exposed to O_3_ treatment. This phenomenon was verified in other species, such as *Populus* sp. [39], and it is possibly related to promoting a new leaf development as a compensative response against O_3_ damage. However, the decrease in the number of leaves was eventually found to be due to O_3_ exposure, which might be related to the potential O_3_ phytotoxicity that triggers programmed cell death, promoting an increase in leaf senescence [40]. When combined with drought stress, the effect can be more substantial once the lack of water and nutrients promotes a decrease in new leaf development.

Both O_3_ and drought stresses were detrimental to the plant biomass increment in all the oak species. In fact, the reduction of biomass due to drought stress is reported for many species and is related to the reduction of water content, diminished leaf water potential and turgor loss, promotion of stomatal closure, and decreased cell enlargement growth [41,42]. As previously revealed by Alonso et al. [21], drought stress does not protect holm oak from O_3_ effects when considering the whole plant response. However, differences between the species responses must be considered when comparing the species sensitivity. For example, we observed that *Q. robur* and *Q. pubescens* invested more in shoots than in roots when exposed to both stresses, while *Q. ilex* performed the opposite, which might be another strategy of *Q. ilex* indicating a hormetic mechanism of tolerance for increasing conducting tissue and maintaining the water flow. These tolerance mechanisms may be associated with morphological/anatomical adjustments, such as a versatile root system, conservative growth and carbon allocation patterns, and diverse adaptations in the leaf morphology [20,43]. This might increase the apoplastic water fraction [44] and promote the species tolerance to O_3_ and drought stress.

### 4.3. Effects of Ozone and Drought Stress on Physiological Parameters

The O_3_ and drought stress negatively affected the physiological parameters. Drought stress induced a decrease of *P*_n_ regardless of the different species, as confirmed by a negative relationship with CDI. A decrease in *P*_n_ with increasing POD_1_ was verified for both sensitive species (*Q. robur* and *Q. pubescens*), while no such reduction was found in *Q. ilex*. The present discussion is based on the species responses to POD_1_ once the flux-based index is more realistic [9]. In fact, AOT40 was positively related to *P*_n_ in the two deciduous oaks, which does not agree with a consensus about O_3_ negatively affecting photosynthetic capacity [45]. In fact, the regression coefficient was very low (=0.000), although the regression slope was numerically significant. Even though data was generated from an underlying distribution, the significance is a rather unlikely biological sense. The data suggest that a biological-sound index such as POD_1_ is superior to AOT40 for the studies of the O_3_ effects on vegetations because it can consider the principal physiological cause of O_3_ damage, i.e., stomatal O_3_ uptake.

Drought stress decreased PNUE and PPUE for all three species, while O_3_ stress negatively affected PNUE for *Q. pubescens* and PPUE for the sensitive species *Q. robur* and *Q. pubescens*. Drought stress is directly related to changes in the allocation of N and P to leaves, no matter the species sensitivity to O_3_ stress. However, a reduced allocation of N and P to the photosynthetic apparatus [5,6,46] is more pronounced in O_3_ sensitive species. The N-uptake efficiency and leaf N efficiency are important traits to improve growth under drought [47]; thus, the decline in root biomass might explain the decrease in PNUE and PPUE for those species, once reduced quantity of absorptive roots reduces water and nutrient uptake as verified for the same oak species in a previous study [48].

### 4.4. Is the Ozone Visible Injury an Indicator to Explain Biomass Reduction or Physiological Damage in Mediterranean Oaks?

The PCA biplot contains the strength of VFI, physiology, and growth relationships, along with the species-specific sensitivity to drought and O_3_ stress. Relationships between O_3_ VFI and biomass growth were discussed by other authors [49,50]. In the present study, we observed that the vector of O_3_ VFI injury (O_3_S) and total biomass (TB) were crossing at the right angle of each other, suggesting a weak association between these two parameters in Mediterranean oaks. However, the O_3_S vector shows the same direction as those of leaf parameters (number of leaves [N.L] and leaf biomass [LB]) in plants presenting more O_3_ VFI, which may indicate the promotion of carbon allocation to leaves as a compensation response against O_3_ injury. In addition, opposite directions of the vectors were found for O_3_ VFI (O_3_S) and net photosynthesis (*P*_n_), PNUE, and the R/S ratio, highlighting a negative correlation between O_3_ VFI and these parameters. The results indicate that O_3_ VFI was not a direct indicator of biomass reduction under elevated O_3_ in these oaks but provides important insights regarding the impairment of photosynthetic capacity and biomass partitioning to roots. Mediterranean oak species generally develop taproots that grow deep into the soil, enhancing resistance to abiotic stress such as drought [51]. However, small amounts of roots due to O_3_ exposure imply a loss of water and nutrient uptake, suggesting that O_3_ VFI should be considered a bioindicator in forests exposed to the combination of O_3_ pollution and drought.

## 5. Conclusions

We examined O_3_- and drought-induced VFI and their effects on growth, biomass, and physiological parameters by using cumulative indexes and oak species known for showing differential sensitivity to these stressors. The increase in POD_1_ promoted the development of specific O_3_ VFI in the isohydric *Q. robur* and the intermediate *Q. pubescens,* while the anisohydric *Q. ilex* was asymptomatic. In *Q. robur*, the presence of O_3_ VFI was decreased under drought probably because drought-induced stomatal closure reduced O_3_ uptake and thus limited O_3_ damage. However, drought stress aggravated O_3_ VFI in *Q. pubescens*. This result indicates the importance of the protective role of antioxidant activity under the combination of O_3_ and drought, which may be weakened by the combined stress factors and become a dominant factor in species that are not strictly isohydric. On the other hand, the drought VFI was clearly distinguished from the O_3_-induced VFI, and it developed with increasing CDI in *Q. robur* and *Q. pubescens* but not in *Q. ilex*, suggesting a high tolerance of *Q. ilex* to drought stress. Therefore, we suggest using the specific O_3_ or drought VFI as a bioindicator, especially for establishing the onset injury CL. 

We also confirmed that *P*_n_ was decreased progressively with POD_1_ and CDI in the two deciduous oaks, in tandem with PNUE decline, suggesting a cumulative effect of O_3_ and drought on photosynthetic capacity. As a result, both stress factors showed a deleterious effect on the development of VFI and biomass growth. Interestingly, the two deciduous oaks increased the allocation to shoot growth rather than to root growth when exposed to both stresses, while an opposite result was found in *Q. ilex*. The imbalance in carbon allocation to roots may reduce the stability against strong winds and impair water uptake under the warming climate expected in future climate change [52,53].

## Figures and Tables

**Table 1:** Phytotoxic O_3_ dose (POD_1_, mmol m^−2^) and accumulated exposure over 40 ppb hourly concentrations (AOT40, ppm h) calculated at the O_3_ visible foliar injury onset, and Cumulative Drought Index (CDI) calculated at the drought visible foliar injury onset for *Q. robur* and *Q. pubescens*.

Water Regime	O_3_ Treat.	Onset O_3_ Injury				Onset Drought Injury	
		POD_1_		AOT40		CDI	
		*Q. robur*	*Q. pubescens*	*Q. robur*	*Q. pubescens*	*Q. robur*	*Q. pubescens*
WW-treated	AA	12.07	Asymp.	17.78	Asymp.	Asymp.	Asymp.
	×1.2	12.40	15.69	16.41	23.77	Asymp.	Asymp.
	×1.4	11.62	20.46	15.15	33.38	Asymp.	Asymp.
MD-treated	AA	13.02	Asymp.	21.74	Asymp.	6.10	6.52
	×1.2	12.13	18.41	16.41	30.43	6.10	4.04
	×1.4	13.07	20.04	21.59	33.38	4.96	6.10
SD-treated	AA	Asymp.	Asymp.	Asymp.	Asymp.	10.20	10.20
	×1.2	Asymp.	13.13	Asymp.	30.43	10.20	10.20
	×1.4	10.72	12.81	26.23	26.23	10.20	10.20

**Table 2:** Evaluation of O_3_ and drought incidence of visible foliar injury (VFI) along the experimental period for *Q. robur* and *Q. pubescens* exposed to different levels of O_3_ and drought.

	Water Regime	O_3_ Treat./DOY	*Q. robur*						*Q. pubescens*					
			209	218	232	240	247	266	209	218	232	240	247	266
O_3_ VFI incidence	WW-treat.	AA	-	-	11	11	11	50	-	-	-	-	-	-
		×1.2	-	11	44	44	44	44	-	-	-	-	11	11
		×1.4	22	33	33	44	56	56	-	-	-	-	-	22
	MD-treat.	AA	-	-	-	11	22	44	-	-	-	-	-	-
		×1.2	-	22	22	22	44	56	-	-	-	-	-	33
		×1.4	-	-	22	33	33	33	-	-	-	-	-	22
	SD-treat.	AA	-	-	-	-	-	-	-	-	-	-	-	-
		×1.2	-	-	-	-	-	-	-	-	-	-	-	22
		×1.4	-	-	-	-	11	11	-	-	-	-	11	33
Drought VFI incidence	WW-treat.	AA	-	-	-	-	-	-	-	-	-	-	-	-
		×1.2	-	-	-	-	-	-	-	-	-	-	-	-
		×1.4	-	-	-	-	-	-	-	-	-	-	-	-
	MD-treat.	AA	-	-	33	33	50	67	-	-	-	11	11	11
		×1.2	-	-	22	58	67	67	11	11	11	22	33	33
		×1.4	-	44	33	44	50	63	-	-	22	33	33	33
	SD-treat.	AA	22	22	67	78	83	89	22	22	39	67	67	67
		×1.2	11	56	61	78	78	89	11	11	22	33	44	44
		×1.4	33	56	78	78	100	100	44	44	67	78	78	78

**Table 3:** Regression coefficients of the multiple linear regression for the species *Q. robur*, *Q. pubescens*, and *Q. ilex*, considering Cumulative Drought Index (CDI) and phototoxic O_3_ dose (POD_1_) for Model 1, and CDI and accumulated exposure over 40 ppb hourly concentrations (AOT40) for Model 2 as predictor factors, and Growth: Plant height increment (cm), Stem diameter increment (cm), and leaf number increment (N. leaves—n); Biomass: Leaf (g), Shoot (g), Root (g), Total biomass (g), and Ratio root/shoot (Ratio R/S); Visible foliar injury (O_3_ and drought—% of plants); Physiological parameters: Photosynthesis (*P*_n_—µmol m^−2^s^−1^), Photosynthetic nitrogen use efficiency (PNUE—µmol m^−2^s^−1^) and Photosynthetic phosphorus use efficiency (PPUE—µmol m^−2^s^−1^) as dependent parameters. Levels of significance (*p*), intercepts and determination coefficients (R^2^) are shown.

	Parameters		Model 1 (POD_1_, CDI)							Model 2 (AOT40, CDI)						
			POD_1_	*p*	CDI	*p*	Intercept	*p*	R^2^	AOT40	*p*	CDI	*p*	Intercept	*p*	R^2^
*Q. robur*	Injury	O_3_	6.736	***	−0.043	n.s	−64.967	***	0.717	0.002	***	−2−027	***	−20.600	***	0.738
		Drought	4.802	***	5.882	***	−68.059	***	0.887	0.001	***	4.524	***	−31.350	***	0.861
	Growth	Height	0.316	**	0.035	n.s	−1.197	n.s	0.308	0.159	***	−0.041	n.s	−0.213	n.s	0.501
		Diameter	0.032	n.s	−0.051	**	5.182	***	0.323	0.019	n.s	−0.059	***	5.214	***	0.338
		N. Leaves	0.068	n.s	−2.338	***	190.955	***	0.521	0.003	n.s	−2.351	***	191.913	***	0.521
	Biomass	Leaf	0.240	***	0.013	n.s	1.741	n.s	0.404	0.068	*	−0.040	n.s	3.743	***	0.191
		Shoot	−0.043	n.s	−0.176	***	12.517	***	0.404	−0.017	n.s	−0.166	***	12.268	***	0.404
		Root	−0.784	**	−0.568	***	40.098	***	0.508	−0.287	*	−0.391	***	35.097	***	0.476
		Total	−0.588	n.s	−0.732	***	54.357	***	0.428	−0.236	n.s	−0.597	***	51.108	***	0.427
		Ratio R/S	−0.063	***	−0.019	***	2.600	***	0.692	−0.022	***	−0.005	n.s	2.163	***	0.491
	Physiology	*P*_n_	−0.629	***	−0.283	***	14.579	***	0.824	0.000	***	−0.152	**	11.930	***	0.738
		PNUE	−0.569	n.s	−0.307	*	14.724	*	0.558	−0.152	n.s	−0.175	*	10.032	**	0.558
		PPUE	−3.751	*	−1.560	**	82.525	**	0.916	−0.668	n.s	−0.671	*	41.067	*	0.767
*Q. pubescens*	Injury	O_3_	1.827	***	0.645	***	−26.982	***	0.329	0.876	***	0.090	n.s	−16.377	***	0.387
		Drought	1.279	*	3.658	***	−23.673	*	0.787	0.660	*	3.259	***	−17.183	**	0.792
	Growth	Height	0.241	***	−0.001	n.s	−0.472	n.s	0.373	0.115	***	−0.060	*	1.039	n.s	0.371
		Diameter	−0.151	***	−0.052	***	8.020	***	0.485	−0.052	**	−0.017	n.s	6.595	***	0.244
		N. Leaves	−4.901	***	−1.801	**	175.047	***	0.390	−2.011	**	−0.644	n.s	136.376	***	0.283
	Biomass	Leaf	−0.019	n.s	−0.012	n.s	4.183	***	−0.070	−0.001	n.s	−0.008	n.s	3.865	***	−0.075
		Shoot	−0.205	**	−0.130	***	13.137	***	0.511	−0.091	**	−0.081	**	11.687	***	0.474
		Root	−0.054	n.s	−0.223	***	22.085	***	0.366	−0.039	n.s	−0.208	***	22.072	***	0.372
		Total	−0.278	n.s	−0.364	***	39.404	***	0.400	−0.132	n.s	−0.297	***	37.625	***	0.398
		Ratio R/S	−0.022	*	−0.017	***	2.132	***	0.316	−0.009	n.s	−0.012	**	1.955	***	0.277
	Physiology	*P*_n_	−0.370	***	−0.255	***	13.500	***	0.795	0.000	***	−0.179	***	12.290	***	0.771
		PNUE	−0.485	*	−0.286	**	14.671	**	0.741	−0.173	*	−0.172	**	10.622	***	0.757
		PPUE	−2.385	*	−1.389	**	70.714	**	0.954	−0.575	n.s	−0.788	**	41.724	**	0.901
*Q. ilex*	Injury	O_3_														
		Drought														
	Growth	Height	0.127	n.s	0.004	n.s	15.423	***	−0.050	0.051	n.s	−0.026	n.s	15.623	***	−0.357
		Diameter	0.153	**	0.031	*	0.799	n.s	0.305	0.042	**	−0.003	n.s	1.483	***	0.229
		N. Leaves	3.238	**	1.194	**	17.246	n.s	0.329	1.218	***	0.445	n.s	23.955	**	0.468
	Biomass	Leaf	−0.151	n.s	−0.035	n.s	5.825	***	0.022	−0.029	n.s	−0.002	n.s	4.846	***	−0.037
		Shoot	0.011	n.s	−0.053	n.s	7.619	***	0.158	−0.000	n.s	−0.055	*	7.750	***	0.157
		Root	0.453	**	0.064	n.s	7.027	***	0.230	0.072	n.s	−0.031	n.s	10.315	***	0.013
		Total	0.313	n.s	0.024	n.s	20.471	***	0.038	0.043	n.s	−0.089	n.s	22.911	***	−0.005
		Ratio R/S	0.095	*	0.012	n.s	0.158	n.s	0.294	0.022	n.s	−0.008	n.s	0.7669	n.s	0.068
	Physiology	*P*_n_	0.094	n.s	−0.173	***	9.090	***	0.630	0.000	n.s	−0.196	***	9.380	***	0.624
		PNUE	−0.014	n.s	−0.122	**	5.768	**	0.840	−0.014	n.s	−0.118	***	5.939	***	0.846
		PPUE	−3.036	n.s	−1.870	*	96.073	*	0.814	−0.478	n.s	−1.124	*	71.158	*	0.772

* = *p* < 0.05, ** = *p* < 0.01, *** = *p* < 0.001, n.s = not significant.

## Data Availability

The data presented in this study are available on request from the corresponding author. The data are not publicly available due to technical limitations and other restrictions.

## Supplementary Materials

The following supporting information can be downloaded at: https://www.mdpi.com/article/10.3390/plants11141836/s1, Figure S1: Results of the multiple linear regression for shoot/root (Ratio R/S) and Photosynthesis (*P*_n_) parameters; Table S1: Raw data of growth parameters.
