# Supplementary material for: Visible Foliar Injury and Ecophysiological Responses to Ozone and Drought in Oak Seedlings
Source: Plants (Basel). 2022 Jul 13;11(14):1836. doi: 10.3390/plants11141836 (PMC9317710; doi:10.3390/plants11141836)
Supplement: Supplementary file 1 [file plants-11-01836-s001.zip › plants-1760036-supplementary.pdf]

Visible foliar injury and ecophysiological responses to ozone and drought in oak seedlings

Bárbara Baesso Moura[1], Elena Paoletti[1*], Ovidiu Badea[2,3], Francesco Ferrini[4] and Yasutomo Hoshika[1]

1. Institute of Research on Terrestrial Ecosystems (IRET), National Research Council of Italy (CNR), Via Madonna del Piano 10, 50019 Sesto Fiorentino, Italy.

2. "Marin Drăcea“ National Institute for Research and Development in Forestry 128 Eroilor Blvd., Voluntari 077190, Ilfov, Romania

3. "Transilvania" University, Faculty of Silviculture and Forest Engineering, 1, Ludwig van Beethoven Str., Braşov 500123, Braşov, Romania

4. Department of Agriculture, Food, Environmental and Forestry Sciences, Section Woody Plants, University of Florence, 50019 Sesto Fiorentino, Italy.

* corresponding author: elena.paoletti@cnr.it

Supplementary material

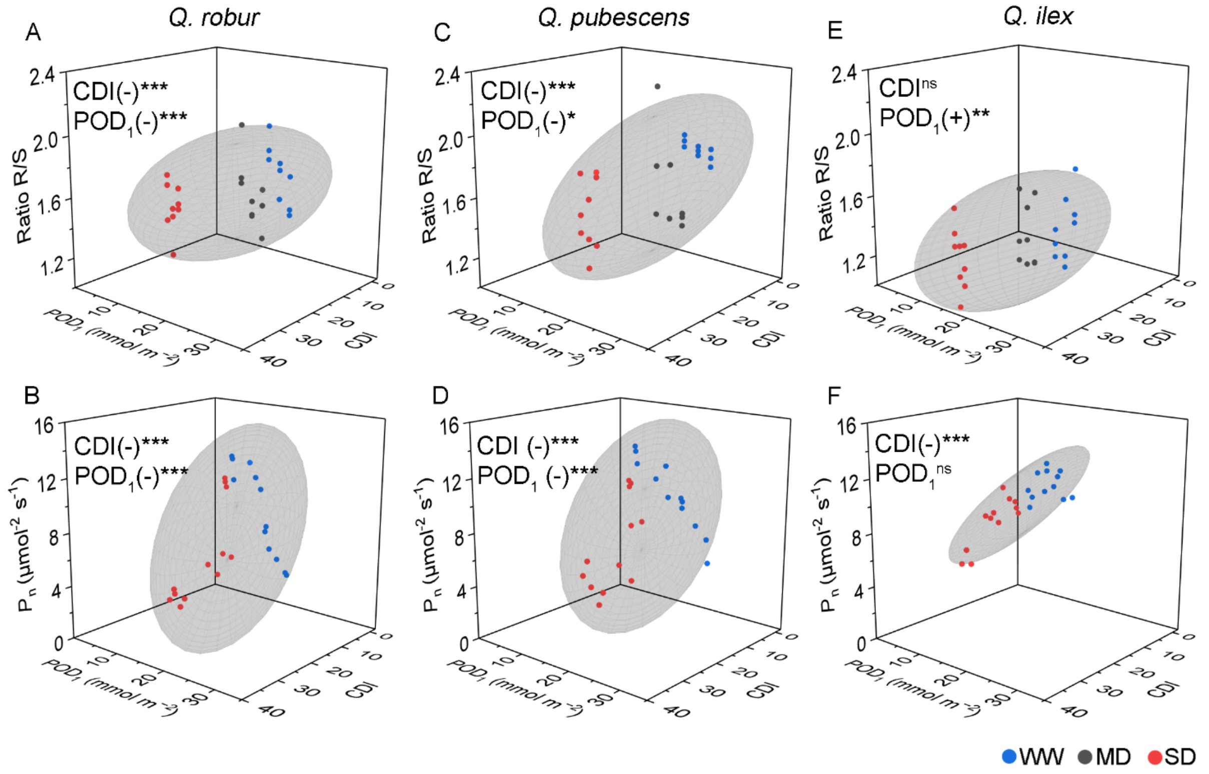

Figure S1. Results of the multiple linear regression, considering Cumulative Drought Index (CDI) and phytotoxic O_3 dose (POD_1) as predictor factors, and shoot/root (Ratio R/S) and Photosynthesis (P_n) as dependent parameters. Colored dots represent well-watered (WW - blue), moderate drought (MD - grey) and severe drought (SD - red). Grey ellipsoids represent a confidence level of 75%. (+) positive regression, (-) negative regression, * = $p < 0.05$, ** = $p < 0.01$, *** = $p < 0.001$, ns = not significant.

Table S1. Growth parameters for the species *Q. robur*, *Q. pubescens,* and *Q. ilex*: Plant height increment (cm), Stem diameter increment (cm), and leaf number increment (N. leaves - n). Biomass parameters: Leaf (g), Shoot (g), Root (g), Total biomass (g), and shoot/root (Ratio R/S). Values are the means ± SE in the different O_3 (AA, ×1.2, ×1.4) and drought (WW, MD, SD) treatments.

**Species**	**Parameter**		**AA**			**×1.2**			**×1.4**		
			WW-treated	MD-treated	SD-treated	WW-treated	MD-treated	SD-treated	WW-treated	MD-treated	SD-treated
Q. ilex	Growth	Height	16.06±1.67	17.39±2.08	15.94±2.51	18.33±3.61	15.50±1.42	14.50±2.75	16.33±2.50	17.33±3.06	18.39±2.36
		Diameter	2.16±0.39	2.31±0.26	2.37±0.64	3.48±0.24	2.28±0.42	2.06±0.37	2.77±0.50	2.32±0.43	3.32±0.36
		N. leaves	42.1±8.23	57.3±6.57	47.2±9.56	59.3±6.29	56.6±1.71	72.7±11.59	51.28±6.32	85.50±14.91	72.0013.28
	Biomass	Leaf	4.45±0.18	4.04±0.23	3.32±0.18	4.04±0.93	3.26±0.64	5.04±0.95	3.59±0.47	4.36±1.60	3.86±0.11
		Shoot	7.48±0.61	7.82±0.31	6.08±0.20	8.48±1.29	6.78±0.75	6.58±1.09	7.58±0.05	6.90±0.54	6.58±0.57
		Root	13.0±0.78	12.4±0.63	12.5±0.95	12.5±1.86	11.5±0.69	10.9±1.45	12.9±0.91	12.2±1.46	10.8±0.68
		Total	24.89±0.26	24.22±0.80	21.92±0.54	25.06±3.91	21.52±2.05	22.52±3.45	24.06±1.35	23.45±3.57	21.21±1.15
		Ratio R/S	1.03±0.03	1.04±0.07	1.40±0.26	1.02±0.10	1.21±0.05	0.96±0.05	1.18±0.05	1.11±0.08	1.08±0.02
Q. pubescens	Growth	Height	2.45±0.2	2.18±0.42	2.73±0.25	2.42±1.34	3.94±0.77	3.15±0.21	6.72±1.15	5.13±0.81	1.5±0.36
		Diameter	5.82±0.77	4.26±0.7	5.04±0.51	5.57±0.28	5.31±0.71	5.08±0.62	4.79±0.38	4.28±0.61	4.88±0.54
		N. leaves	88.3±14.5	60.89±7.75	74.67±12.2	85.1±4.86	120±12.2	62.1±0.92	39.8±3.29	82.7±16	59.8±7.24
	Biomass	Leaf	3.43±0.79	3.01±0.59	3.65±0.36	3.77±0.09	4.48±0.85	3.29±0.71	3.04±0.29	5.23±1.12	3.59±0.32
		Shoot	9.84±0.81	9.17±1.36	7.19±0.23	8.17±0.25	9.37±0.49	7.92±0.51	9.02±1.10	8.28±0.88	6.64±0.47
		Root	21.6±1.16	20.9±1.29	16.4±1.98	19.3±0.80	20.9±0.74	14.5±0.81	21.9±2.70	18.6±2.52	16.4±2.51
		Total	35.1±0.97	33.1±1.87	27.2±2.47	31.6±0.34	34.7±1.19	25.7±1.76	33.9±3.91	32.1±4.37	26.6±3.23
		Ratio R/S	1.82±0.04	1.77±0.44	1.5±0.21	1.77±0.03	1.48±0.21	1.32±0.23	1.74±0.06	1.38±0.04	1.59±0.27
Q. robur	Growth	Height	2.78±1.8	2.52±0.69	1.6±0.61	2.35±0.63	3±1.5	2.03±0.57	5.48±1.77	6.2±0.61	4.67±1.76
		Diameter	5.62±0.55	4.3±1.2	5.06±0.52	6.18±0.55	4.79±0.71	3.29±0.37	5.76±0.41	5.73±0.42	4.64±0.65
		N. leaves	187±26.8	166±17.3	134±11.1	190±17.8	166±4.86	143±17.5	177±13.9	208±35	117±12
	Biomass	Leaf	4.93±0.86	5.15±0.51	4.58±0.22	5.25±0.67	5.46±0.61	4.41±0.46	6.17±0.63	5.90±1.55	3.93±0.26
		Shoot	12.5±1.24	12.4±2.46	7.71±0.24	11.6±1.86	10.4±1.18	7.62±0.50	10.6±2.21	9.66±2.12	7.21±0.85
		Root	29.2±6.04	26.2±2.32	19.8±0.63	26.9±3.59	21.9±2.06	16.2±1.50	25.3±4.55	24.7±4.74	18.8±1.56
		Total	46.6±8.09	43.8±4.94	32.1±0.77	43.7±6.05	37.8±3.85	28.3±1.69	42.0±7.35	40.3±8.15	29.9±1.78
		Ratio R/S	1.78±0.12	1.71±0.22	1.58±0.16	1.56±0.14	1.39±0.06	1.37±0.17	1.42±0.15	1.41±0.17	1.55±0.07
